# The prevalence, risk factors and outcomes of anaemia in South African pregnant women: a protocol for a systematic review and meta-analysis

**DOI:** 10.1186/s13643-020-01460-0

**Published:** 2020-09-11

**Authors:** Vinogrin Dorsamy, Chauntelle Bagwandeen, Jagadesa Moodley

**Affiliations:** 1 Laboratory Medicine and Medical Sciences, College of Health Sciences, University of KwaZulu-Natal, KwaZulu-Natal, South Africa; 2 Discipline of Public Health Medicine, College of Health Sciences, University of KwaZulu-Natal, KwaZulu-Natal, South Africa; 3 Women’s Health and HIV, School of Clinical Medicine, University of KwaZulu-Natal, KwaZulu-Natal, South Africa

**Keywords:** Anaemia, Pregnancy, South Africa

## Abstract

**Background:**

A significant cause of morbidity and mortality during pregnancy is maternal anaemia. The causes of anaemia and the sequelae are varied, and the prevention and management are public health challenges, especially in resource-limited settings and certain geographic locations. South Africa is plagued by a quadruple burden of disease, with high maternal mortality rates affected by hypertensive disorders of pregnancy, HIV, tuberculosis and neglected tropical diseases. This is most prevalent in people of lower socio-economic status. Poor nutrition, chronic infections, lack of access to health care facilities and poor compliance with micronutrient supplementation all contribute to maternal anaemia. The aim of this study is to systematically map the literature to ascertain the pooled prevalence and associated causes of anaemia in the South African pregnant population, which will enable health care workers and other key stakeholders to more pertinently address Sustainable Development Goal 3 focussing on good health and reducing maternal mortality.

**Methods:**

PubMed, CINAHL, EMBASE, EBSCO, Ovid maternity and infant care databases, Cochrane Database of Systematic Reviews, Web of Science and SCOPUS will be searched using the keywords ‘anaemia’, ‘haemoglobin’, ‘pregnancy’, and ‘South Africa’ to conduct a systematic review and meta-analysis to explore, describe and map literature on the distribution and burden of anaemia in pregnant women in South Africa. The reference list of articles selected for review will be scanned for other articles of interest to our study question. Studies published in any language will be included in this review. As there may be differences in sampled populations in South Africa based on geography and sociodemographic factors, a weighted inverse-variance meta-analysis using a random-effects model will be carried out to generate a pooled prevalence estimate. A Funnel plot and Egger’s regression test will be conducted to assess publication bias. Heterogeneity among studies will be checked using *I*^2^ to determine dispersion and meta-regression analysis will be performed to investigate the source of heterogeneity. The articles obtained by these searches will be analysed for causative factors, severity and outcomes by a parallel and independent review team, using suitable eligibility criteria. Screening, data extraction and quality appraisal will be conducted independently by two authors. Disagreement will be resolved by independent assessment by a third reviewer. Sub group analysis by region, stage of pregnancy, socio-economic status, severity and cause of anaemia will be conducted if sufficient data is available. Data will be analysed using statistical software and presented in evidence tables and in meta-analytic forest plots.

**Discussion:**

This protocol is developed to systematically review the literature on the prevalence and severity of anaemia, risk factors and outcomes in pregnant women in South Africa. Correlation of factors contributing to the development of anaemia and other disorders during pregnancy will facilitate exploration of appropriate medical and behavioural change interventions implemented within other countries or regions that mitigate risk. This study will assist local health systems to inform public health policies and practises for more favourable maternal and fetal outcomes.

**Trial registration:**

This protocol is registered with PROSPERO (CRD42020157191)

## Background

Anaemia, defined as a decrease in red blood cells or haemoglobin, or the reduced capacity of red blood cells to carry oxygen [1] is a common chronic problem in low- to middle-income countries, such as South Africa. It is a product of socio-economic disparity, affecting mostly the poor and less educated [2]. It is most prevalent in the vulnerable: children, adolescents in their growth phase and females of reproductive age [3]. The condition is exacerbated by pregnancy when there is an increased burden to deliver oxygen to the fetus and can result in intrauterine growth restriction, reduced iron for the baby and increased maternal and perinatal morbidity and mortality [4].

Anaemia affects almost 2 billion people worldwide, with iron deficiency anaemia being the commonest cause. As with other low- and middle-income countries with similar sociodemographic characteristics, the South African population is more susceptible to anaemia due to the prevalence of chronic nutritional insufficiency and concomitant infective conditions [5]. The World Health Organisation (WHO) reports the prevalence of anaemia in women of reproductive age women in sub-Saharan Africa to be 57.1% [6], but this varies between regions, with the highest in Central and West Africa (61%) and the lowest level reported in Southern Africa (34%) in 2011. While the authors report a declining trend from 1995 to 2011, current prevalence data for the region is not available, and data for anaemia in pregnant South African women is outdated. The last census type study was conducted in 2012, and more current studies report on isolated sites only.

Food fortification and supplements during gestation are the mainstay of prophylaxis against the sequelae of iron deficiency [7]. However, anaemia is also due to other causes. In South Africa, pregnant women face the compound burden of infective causes of anaemia, such as HIV and TB, neglected tropical diseases, like hookworm and schistosomiasis, other bacterial infections, as well as nutritional insufficiency and inherited haemoglobinopathies and thalassaemias, which may also exacerbate negative pregnancy outcomes [6].

However, given that physiological anaemia in pregnancy is associated with protection against fulminant expression of infections such as schistosomiasis and other parasitaemias, non-discriminatory mass treatment with iron supplementation in these cases may contribute adversely to maternal and fetal morbidity and mortality [8].

It is therefore important to determine the current burden, causes and consequences of anaemia in pregnant women so as to better guide appropriate treatment protocols which will focus on the specific cause of anaemia and may differ to current recommendations.

## Aim and objectives

It is the purpose of this systematic review to establish the present pooled prevalence of anaemia in South African pregnant women. The specific objectives are to

- Determine the causal or associated factors of anaemia in the pregnant population
- Describe the type and severity of anaemia
- Describe the fetal and maternal outcomes associated with anaemia in pregnancy

Correlation of factors contributing to the development of anaemia and other disorders during pregnancy will facilitate exploration of medical interventions as well as inform behavioural changes to mitigate current and future risk.

## Methodology

This protocol is written in line with the recommendations of the Preferred Reporting Items for Systematic Reviews and Meta-analysis for Protocols (PRISMA-P) guidelines [9]. The results will be reported based on the PRISMA 2009 statement, and article screening and selection process will also be demonstrated through a PRISMA-P flow diagram.

### Eligibility of research question

The eligibility of the research question was determined by the Population Exposure Comparator Outcomes (PECO) framework as illustrated in Table 1.

**Table 1:** PECO framework for eligibility of research question

Population	Pregnant women with anaemia in South Africa
Intervention/exposure	Haemoglobin concentration < 10 g/dL
Comparator	Pregnant women without anaemia in any trimester
Outcomes	Prevalence, severity, risk factors, maternal morbidity and mortality, fetal outcomes

All studies reporting on anaemia and reporting on prevalence or haemoglobin concentration during pregnancy in a South African population will be included. There will be no restriction on study design, setting or date of publication. Articles published in any language will be accepted and translated into English using Google Translate if possible. If it is not possible to translate abstracts into English, they will be removed from the study, but listed in an appendix.

### Identifying relevant studies

Search terms used and their synonyms were identified using the Medical Subject Headings (MeSH). The uniterms and Boolean operators in English to be used in the search strategies are (anemia OR anaemia OR haemoglobin OR hemoglobin OR haematocrit OR hematocrit) AND (Pregnancy OR Pregnant women OR Gravidity OR Maternal exposure OR Mother OR Pregnant OR Gravid OR Obstetric OR Antenatal OR Antepartum OR Gestation) AND (South Africa OR Southern Africa or South African or Sub-Saharan Africa). The search strategy will be adapted for the other electronic databases to be searched.

The search strategy was piloted in November 2019 to check the appropriateness of selected electronic databases and key words as depicted in Table 2.

**Table 2:** Search strategy piloted in PubMed

Database used	Search terms	Articles found
PubMed	(((((((((“anaemia”) OR “anemia”) OR “haemoglobin”) OR “hemoglobin”) OR “iron deficiency”) OR “haematocrit”) OR “hematocrit”)) AND (((((((((((“pregnancy”) OR “pregnant”) OR “maternal”) OR “mother”) OR “gravid”) OR “maternal exposure”) OR “obstetric”) OR “antenatal”) OR “antepartum”) OR “gestation”) OR “gestational”)) AND (((“south africa”) OR “southern africa”) OR “south african” OR “Sub-Saharan”)	486

### Study selection

The eligibility of retrieved studies will be assessed using the following criteria:

Inclusion criteria

For studies to be included they should meet the following criteria:

- Be available in full text
- Must include pregnant women living in South Africa
- Must focus on anaemia in pregnancy using the WHO classification of anaemia: < 10 mg/dL Hb or include such data on pregnant women in studies ascertaining prevalence in various population groups

Exclusion criteria

Studies will be excluded should they be:

- Not available in full text
- Not reporting anaemia or haemoglobin concentrations in pregnancy
- Full papers not available despite requests to the authors

An abstract screening tool, using Google Forms, will be developed and distributed to the researchers. Abstract screening, followed by full article screening, will be conducted manually, including those articles for which an abstract is not available.

A citation manager Zotero v5.0.81 (Zotero.org) will be used to create a library for this review. The primary investigator (VD) will conduct a search using the key fields in the databases created. Eligible studies will be exported to the citation manager, with duplicates removed before abstracts are screened by two authors/investigators (VD and CB). A test of interrater reliability using Cohen’s Kappa will be performed with a cut-off point of .80 (indicating substantial agreement). If this precision is not met, all discrepancies in selected abstracts will be evaluated by the third author/investigator (JM) for eligibility. Full article screening guided by the eligibility criteria will then be carried out independently. A subject librarian will be consulted to optimise the article search procedure to help with retrieving and finding articles to be included in the full-article screening. For those papers that are electronically unavailable, authors will also be contacted directly. The screening results will be reported using a PRISMA-P flowchart [9].

### Charting of data items

As illustrated in Table 3 below, a data charting table will be used to extract background information and process the data items from each study selected. To ensure that all pertinent information regarding the relevant aspects for the study is collected, a data charting form will be developed and piloted, and continually updated.

**Table 3:** Data charting table

Author and date	
Study title	
Journal full reference	
Sampling method	
Study design	
Theoretical framework	
Data collection methodology	
Data analysis	
Average age	
Gravidity	
Socioeconomic status	
Educational level: primary/secondary/tertiary	
Geographic location	
Development at location: urban/semi urban/rural	
Province	
Prevalence of anaemia Hb concentration	
Incidence of anaemia Cause of anaemia, list if multiple	
HIV status (report percentages + and −)	
Tb	
Other co-morbidity (if any)	
CD 4 count	
Viral load	
ARV’s TB Other co-morbidity ( if any)	
Supplements	
Present pregnancy: gestational age	
Previous pregnancy/ies	
Live birth	
Pregnancy induced hypertension	
Eclampsia Birth outcomes Fetal morbidity/mortality	
Same partner	
Most relevant finding	
Conclusion	
Comments	

### Collating, summarising and reporting the results

#### Risk of bias and quality assessment

The quality and risk of bias of selected studies will be performed by both reviewers using the Hoy tool [10] which determines the risk of bias in prevalence studies. The tool uses 10 criteria to assess both internal and external validity of studies based on participant selection criteria as a representation of overall population, how information is collected, and whether parameters used to determine prevalence was consistent in each study amongst participants [10]. An overall risk of bias will then be determined based on the individual scores obtained for each of the 10 items and graded according to the number of high risks found: low (≤ 2), moderate (3, 4) and high (≥ 5). Where information related to the 10 items is not available, it will be captured as high risk.

The quality and scientific evidence of the studies will be determined using the Grading of Recommendations Assessment, Development and Evaluation (GRADE) tool to assess risk of bias, imprecision, inconsistency and indirectness, as well as publication bias, and placed into one of four categories, ranging from very low (where the true effect is probably markedly different from the estimated effect) to very high (that which was observed is most likely to be true). Randomised controlled trials without limitations will therefore present the most reliable evidence, with observational studies falling into the lowest category, given the potential for confounding. The strength of the recommendations emanating from these studies will be assessed based on the quality of evidence presented. The two authors (VD and CB) will independently assess the quality of the studies using the data extraction tool, with consensus on disagreement being achieved with the assistance of a third author (JM).

#### Data synthesis and analysis

Heterogeneity among studies will be checked using *I*^2^ to determine dispersion. As there may be substantial heterogeneity based on differences in sampled populations in South Africa based on geography and sociodemographic factors, a weighted inverse-variance meta-analysis using a random-effects model will be carried out to generate a pooled prevalence estimate. A Funnel plot and Egger’s regression test will be conducted to assess publication bias. The articles obtained by these searches will be analysed for causative factors and outcomes by a parallel and independent review team, using suitable eligibility criteria. Screening, data extraction, and quality appraisal will be conducted independently by two authors (VD and CB). Disagreement will be resolved by independent assessment by a third reviewer (JM). Sub group analysis by region, stage of pregnancy, socio-economic status, severity, and cause of anaemia will be conducted if sufficient data is available. Data will be analysed using STATA v16 (2019, College Station, TX: StataCorp. LLC.) and presented in evidence tables and in meta-analytic forest plots.

## Discussion

Thus far very few investigations have focussed on anaemia and concomitant disorders during pregnancy in the Southern African region. Active research in this vulnerable population is urgently required to identify the type and burden of anaemia affecting South African pregnant women and its association with contributing factors such as nutrient deficiency, growth and development of the fetus and neonate, co-morbid diseases such as TB and HIV, hypertensive disorders of pregnancy as well as clinical outcomes and long-term sequelae. This protocol has been developed to determine the prevalence, type and causes of anaemia affecting pregnant women in South Africa. The information thus obtained will best inform decision-makers in developing health policies and practises most suitable to positively impact on maternal and fetal outcomes.

## Supplementary information

**Additional file 1.** Prisma P Checklist

## Data Availability

All data generated or analysed during this study will be included in the published systematic review article and will also be available upon request. The Prisma-P checklist is available in Additional file 1.docx

## Abbreviations

WHO: World Health Organisation
HIV: Human immunodeficiency virus
TB: Tuberculosis
PRISMA-P: Preferred Reporting Items for Systematic Reviews and Meta-analysis for Protocols
PECO: Population Exposure Comparator Outcomes
Hb: Haemoglobin
GRADE: Grading of Recommendations Assessment, Development and Evaluation
